# Paternal Factors and Inequity Associated with Access to Maternal Health Care Service Utilization in Nepal: A Community Based Cross-Sectional Study

**DOI:** 10.1371/journal.pone.0130380

**Published:** 2015-06-24

**Authors:** Dharma Nand Bhatta, Umesh Raj Aryal

**Affiliations:** 1 Department of Public Health, Pokhara University, Nobel College, Sinamangal, Kathmandu, Nepal; 2 Faculty of Medicine, Epidemiology Unit, Prince of Songkla University, HatYai, Thailand; 3 Department of Community Medicine, Kathmandu Medical College, Sinamangal, Kathmandu, Nepal; University College London, UNITED KINGDOM

## Abstract

**Background:**

The threat of maternal mortality can be reduced by increasing use of maternal health services. Maternal death and access to maternal health care services are inequitable in low and middle income countries.The aim of this study is to assess associated paternal factors and degree of inequity in access to maternal health care service utilization.

**Methods:**

Analysis illustrates on a cross-sectional household survey that followed multistage-cluster sampling. Concentration curve and indices were calculated. Binary logistic regression analysis was executed to account paternal factors associated with the utilization of maternal health services. Path model with structural equation modeling (SEM) examined the predictors of antenatal care (ANC) and institutional delivery.

**Results:**

The finding of this study revealed that 39.9% and 45.5% of the respondents’ wives made ANC visits and utilized institutional delivery services respectively. Men with graduate and higher level of education were more likely (AOR: 5.91, 95% CI; 4.02, 8.70) to have ANC of their wives than men with no education or primary level of education. Men with higher household income (Q5) were more likely (1.99, 95% CI; 1.39, 2.86) to have ANC for their wives. Similarly, higher household income (Q5) also determined (2.74, 95% CI; 1.81, 4.15) for institutional delivery of their wives. Concentration curve and indices also favored rich than the poor. SEM revealed that ANC visit was directly associated to institutional delivery.

**Conclusions:**

Paternal factors like age, household wealth, number of children, ethnicity, education, knowledge of danger sign during pregnancy, and husband’s decision making for seeking maternal and child health care are crucial factors associated to maternal health service utilization. Higher ANC coverage predicts higher utilization of the institutional delivery. Wealthier population is more concentrated to maternal health services. The inequities between the poor and the rich are necessary to be addressed through effective policy and programs.

## Background

Recent global reports claim that maternal death approximates to 287,000per year, to which 99% accounted in low and middle income countries (LMICs)[1]. However, the data show that the maternal death has declining trends since last three decades among LMICs[2]. Nepal demographic and health survey (NDHS) showed that the maternal mortality ratio (MMR) per 100,000 live birthshas declined from 539 in 1996 to 281 in2006 in Nepal[3]. A recent results from UN studyestimated170 MMR in 2010 in Nepal[1]. The data show that Nepal has achieved the target of reducing MMR set by Millennium Development Goal, and this achievement of Nepal is a result of the increased number of skilled birth attendants (SBA)and door to door service of female community health volunteers (FCHV) in recent years. Among the South Asian countries Pakistan and Afghanistan have higher MMR[2,4].

A number of women die every year during pregnancy and childbirth due to the lack of access to SBA in LMICs[1,5]. Antenatal care (ANC) has helped to reduce the risk of maternal deaths caused by complications during pregnancy[6]. Although, the comparative contribution of ANC to maternal health are contradicted to one another[6,7], a systematic review has identified a significant association between the ANC and the utilization of institutional delivery services [8].A declining trend of maternal mortality has been observed in few South Asian and other LMICS, and it has helped to systematize and improve the strategies to increase the number of SBA and the use of ANC services[9–13].

Findings of different studies revealed that delivery by SBA, ANC and institutional delivery had a strong association with urban residence, husband’s education, age and income[3,14–16]. In addition, consent for seeking treatment, location of health facility, and not wanting to go alone were also associated to maternal health care service utilization in Nepal [3]. Furthermore, low staffing, weak referral system, unavailability of equipment and drugs were the potential reasons for lower utilization of maternal health services [10]. Decision making power of women, decisions made by mother-in-law, husbands and other parents were found to be other crucial determinants for low utilization of available institutional delivery care, SBA and ANC [15,17]. Moreover, financial constrains and wealth were also significant determinants of utilization of maternal health services[9,18].

Household economic status, ethnicity, and gender are few structural factors that results to health inequality, and are associated with each other [19,20]. Findings from LMICs revealed the access to maternal health care services was determined among the rich and maternal death was more rampant among the poor[21,22]. Considerable inequalities exist in the utilization of institutional delivery services, births with SBAand ANC [14,15,23]. Another study from fifty four countries revealed that the SBA exposure was less equitable indicator [24]. Inequity challenges attempts to continue enhancements across all fragments of the world in accessing improved maternal health facilities[25]. Increasing use of maternal health care services among the poor is ensured only through adequate and quality health care services [26].Equal access of maternal health services is an equal need for all the pregnant and/or mothers.

Literatures related to paternal factors and equity in the access to maternal health care services utilization is sparse. Therefore, the objective of this paper was to assess different paternal factors and inequalities associated with access to maternal health services utilization in order to help the programmers, and the policymakers to recognize the needs to enhance quality and equitable maternal health services in Nepal.

## Methods

### Study design and participants

A community based cross-sectional survey was conducted in Kathmandu district situated in the central region of Nepal. The study was carried out between May and December 2010. The units of analysis for this study were male of a household head whose wife had given birth to at least one child[27]. The age range of male was between 20 and 55 years.

Of the 57 village development committees (VDCs), one municipality and one metropolitan in the district, 20 VDCs and one metropolitan were selected randomly to include in this survey. A simple random sampling method was used to select 20 VDCs and wards of the metropolitan. A cluster sampling technique was used to identify the study population. Total 21 wards from 20 VDCs and one ward of the metropolitan were selected as the cluster by probability proportionate to the number of households. One cluster consisted of 50 to 300 households, whereas the wards had more than 400 households which were further divided into sub wards[27]. If wards had less than 50 households, they were merged with neighboring wards to make one cluster with 50 and 300 households[27]. Ultimately, 2200participants, that consisted of 100 household heads from 20 VDCs and 200 household heads from metropolitan were selected randomly for interview[27]. Overall response rate was 99% and remaining of the participants refused to participate in the study. Household heads were selected after a random initiate at a central part in the cluster. If the household head were absent at the time of the survey and were not as per the inclusion criteria, the household head next to the household was included for the interview.

### Interview process

A structured questionnaire was administrated through face-to-face interview by trained enumerators. The questionnaire was first developed in English and then later translated into Nepali. Necessary changes had been made after the pre-testing of the questionnaires.

Written informed consent was taken from all the participants before administering the survey questionnaires. Thumb print was obtained from those participants who were unable to write. Respondent’s name was not used to ensure confidentiality of the information. Right to withdraw at any point of the time during study was informed to respondents. The study activities were approved by institutional ethical review committee of Nobel College, Pokhara University (Ref. 031, April 04, 2010).

### Study variables and measures

It shows that the definition of the service utilization related to maternal health, where response was ‘yes’ that could be the access or utilization of service.

Questions were developed as:

Did your wife have ANC visit during her last pregnancy?

Did your wife give birth to her last child at any health (facility) institution?

Did you make arrangement of SBA for your wife’s last delivery?

All answers coded as ‘**no = 0’ and ‘yes = 1’.** ANC visits and institutional delivery were taken as outcome variables.

Other independent variables’ information were collected inquiring on age; education status{coded as illiterate and primary level (cannot read and write to class five), secondary to higher secondary level (received education from class six to class twelve), graduate and above level (received education more than class twelve)}; monthly household income (all the amount of income earned by family members); ethnicity (indigenous and non-indigenous); employment status (formal occupation: one had job in government and non-government, and formally recruited or established, non-formal occupation: not formally recruited or had no formal appointment letter in any sector); religion (Hindu or other than Hindu), and the number of living children.

Knowledge of danger sign during pregnancy of wife was asked to the respondents. Inquiry of presence of any of the given conditions like: fever, edema, bleeding, convulsion, severe headache and infection were asked to the respondents. The answers were coded as ‘yes = 1’ or ‘no = 0’.Decision maker to receive maternal and child health care was measured using a question; who make decision to receive maternal and child health care in your home? The answer was coded as ‘wife = 0’, ‘husband or himself = 1’, ‘his mother = 2’, and ‘his father = 3’. Monthly household incomes were coded as first quintile (Q1), second quintile (Q2), third quintile (Q3), fourth quintile (Q4), and fifth quintile (Q5) respectively.

### Analysis

#### Concentration Index (CI)

The equity gap was presented showing the difference between lowest and highest quintiles. The equity ratio was calculated dividing highest quintile by the lowest. The ratio was used to indicate the inequality in maternal health services use among the economic status of the population. Household economic status was categorized with respective quintile groups (Q1 = represents poorest to Q5 = represents richest) by ranking household income. The concentration curve plotted the cumulative proportion of maternal health related outcomes ranked by household income quintile against the cumulative sample population. The ideal equality has been reflected when the concentration curve overlapped with the diagonal line of equality. Concentration curve above the diagonal line of equality indicated unequal maternal health service use among poor people and concentration curve below the diagonal line of equality indicated unequal maternal health service use among rich people[28,29]. Concentration index was projected as twice the area between the line of equality, and the concentration curve ranged between -1 to 1. Positive value showed the rich people (pro-rich) having better coverage than poor people (pro-poor) and zero indicated the perfect equity[28,29].

#### Path analysis with structural equation modeling

Structural equation model was analyzed to show the associations between the dependent and independent variables. The goodness-of-fit of the models was evaluated with the Root Mean Squared Error of Approximation (RMSEA), Comparative Fit Index (CFI), Tucker-Lewis Index (TFI) and likelihood ratio of chi-square. The RMSEA values less than or equal 0.05 reflects the good fit of the model. CFI and TFI values more than or equal .95 are desirable for good fit of the model indicating that the model reproduces 95% of the co-variation in the data. Lagrange multiplier test was used to improve model fit and establish additional relationships. Finally, the stability of the model was checked. The path model included dependent and independent variables positioned the age, education, ethnicity, occupation, and number of children as predictors of the intermediate variables of income, ANC, and institutional delivery, further income in turn predicted the outcome of ANC and institutional delivery utilization. Bi-directional causality between ANC and institutional delivery were also assessed.

Both univariate and multivariate analysis were applied to identify factors associated with maternal health service utilization. In univariate analysis, unadjusted odds ratio was computed to measure association between ANC and other independent variables including institutional delivery. Multivariate analysis was done with logistic regression to determine associations. All the explanatory variables like age, income, education, occupation, religion, ethnicity, number of children, decision maker for seeking maternal and child health and knowledge about danger sign were adjusted in multivariate logistic regression analysis. The likelihood ratio test was done to assess qualified involvement of terms entering into the model with a P-value of more than 0.05 considered as the cut-point for removing variables in a stepwise fashion. Multicollinearity among independent variables in the logistic regression was determined by examining variance inflation factor (VIF). Binary logistic regression output was summarized with crude and adjusted odds ratios (AOR) with 95% confidence interval (CI). Stata version 12.0 and R software was used for data analysis.

## Results

More than one third (38.8%) of the respondents were of the age more than or equal to 35 years, and majority (56.2%) of the respondents under wealthiest quintile (Q5) were belong to the age equal to or more than 35 years. A ratio of the two (Q1 and Q5) quintiles was above 1.0 in this age group. Men who had graduate and higher level of education have more gaps in equity and equity ratio among wealthiest and poorest quintile. The findings revealed that the ratio of the richest (Q5) and the poorest (Q1) quintile was 1.73 with SBAs, 1.84 with ANC and 1.79 with institutional delivery (Table 1).

**Table 1:** Characteristics of the respondents (N = 2178).

Characteristics	n	%	Q1 (%) poorest	Q2 (%)	Q3 (%)	Q4 (%)	Q5 (%) richest	Ratio Q5/Q1	Equity Gap (Q5-Q1)	(Q2-Q1)/ (Q5-Q4)
**Age**										
≤24 years	315	14.5	16.9	21.3	16.7	10.5	4.2	0.25	-12.7	-0.70
25–34 years	1017	46.7	37.3	52.5	55.6	52.6	39.6	1.06	2.3	-1.17
≥35years	846	38.8	45.8	26.2	27.8	36.8	56.2	1.23	10.4	-1.01
**Education**										
Illiterate & primary level	666	3.6	45.8	47.5	30.6	13.2	4.2	1.09	-41.6	-0.19
Secondary to higher secondary level	891	40.9	20.3	50.8	58.8	57.9	27.1	1.33	6.8	-0.99
Graduate & above level	621	28.5	33.9	1.6	1.6	28.9	68.8	2.03	34.9	-0.81
**Occupation**										
Informal employment	1143	52.5	91.5	62.3	41.7	21.1	25	0.27	-66.5	-7.49
Formal employment	1035	47.5	8.5	37.7	58.3	78.9	75	8.82	66.5	-7.49
**Religion**										
Others (Buddhist, Christian, Muslim)	279	12.8	10.2	21.3	19.4	5.3	6.2	0.61	-4.0	12.33
Hindu	1899	87.2	89.8	78.7	80.6	94.7	93.8	1.04	4.0	12.33
**Ethnicity**										
Indigenous	405	18.6	16.9	24.6	25	13.2	12.5	0.74	-4.4	-11.00
Non-indigenous	1773	81.4	83.1	75.4	75	86.8	87.5	1.05	4.4	-11.00
**Number of children**										
1–2 children	1476	67.8	57.6	59.0	75.0	78.9	77.1	1.34	19.5	-0.78
≥ 3 children	702	32.2	42.4	41.0	25.0	21.1	22.9	0.54	-19.5	-0.78
**Knowledge of danger signs**										
No	1593	73.1	66.1	88.5	75	73.7	60.4	0.91	-5.7	-1.68
Yes	585	26.9	33.9	11.5	25	26.3	39.6	1.17	5.7	-1.68
**Decision maker for MCH**										
Wife	90	4.1	3.4	3.3	2.8	2.6	8.3	2.44	4.9	-0.02
Husband	1935	88.8	86.4	88.5	88.9	89.5	91.7	1.06	5.3	0.95
Mother	126	5.8	8.5	8.2	2.8	7.9	0.0	0.0	-8.5	0.34
Father	27	1.2	1.7	0.0	5.6	0.0	0.0	0.0	-1.7	∞
**SBAs**										
**No**	1134	52.1	54.2	78.7	50.0	47.4	20.8	0.38	-33.4	-0.92
**Yes**	1044	47.9	45.8	21.3	50.0	52.6	79.2	1.73	33.4	-0.92
**Place of ANC**										
No ANC	1323	60.7	66.1	70.5	69.4	57.9	37.5	0.57	-28.6	-0.21
Institutional	855	39.3	33.9	29.5	30.6	42.1	62.5	1.84	28.6	-0.21
**Place of delivery**										
Home delivery	1188	54.5	55.9	80.3	55.6	52.6	20.8	0.37	-35.1	-0.77
Institutional delivery	990	45.5	44.1	19.7	44.4	47.4	79.2	1.79	35.1	-0.77

Men aged greater than or equal to 35 years were likely (AOR 4.38, 95% CI; 2.99, 6.43) to have more ANC of their wives than men who aged less than or equal 24 years. Men with graduate and higher level of education were more likely (5.91, 95% CI; 4.02, 8.70) to have ANC of their wives than the men who were uneducated or had primary level of education. Men who had higher household income (Q5) were more likely (1.99, 95% CI; 1.39, 2.86) to have ANC of their wives than those who had lower household income (Q1). Men with knowledge on danger signs during pregnancy (4.47, 95% CI; 3.43, 5.81) were more likely to have ANC of their wives than men with no knowledge about danger signs during pregnancy. Men who had equal to or more than three children (0.74, 95% CI; 0.57, 0.97) were less likely to have ANC of their wives than the men who had equal to or less than two children (Table 2).

**Table 2:** Paternal factors associated with institutional ANC care of wife (N = 2178).

Characteristics	Crude odds ratio 95% CI	P-value	Adjusted odds ratio 95% CI	P-value
**Age**				
≤24 years	Reference		Reference	
25–34 years	1.98 (1.50, 2.63)	<0.001	2.62 (1.88, 3.67)	<0.001
≥35years	2.14 (1.60, 2.85)	<0.001	4.38 (2.99, 6.43)	<0.001
**Education**				
Illiterate & primary level	Reference		Reference	
Secondary to higher secondary level	3.42 (2.65, 4.40)	<0.001	2.66 (2.30, 3.55)	<0.001
Graduate & above level	12.23 (9.32, 16.06)	<0.001	5.91 (4.02, 8.70)	<0.001
**Income**				
Q1-poorest	Reference		Reference	
Q2	0.82 (0.63, 1.05)	0.121	2.11 (1.50, 2.96)	<0.001
Q3	0.86 (0.64, 1.15)	0.312	1.32 (0.89, 1.95)	0.164
Q4	1.42 (1.07, 1.87)	0.014	1.63 (1.11, 2.39)	0.013
Q5-richest	3.25 (2.49, 4.23)	<0.001	1.99 (1.39, 2.86)	<0.001
**Number of children**				
1–2 children	Reference		Reference	
≥ 3 children	0.53 (0.44, 0.65)	<0.001	0.74 (0.57, 0.97)	0.031
**Knowledge of danger signs**				
No	Reference		Reference	
Yes	7.02 (5.68, 8.68)	<0.001	4.47 (3.43, 5.81)	<0.001
**Decision maker for MCH**				
Wife	Reference		Reference	
Husband	0.68(0.44,1.04)	0.073	1.20 (0.74, 1.95)	0.463
Mother of respondent	0.27(0.15,0.49)	<0.001	0.92 (0.46, 1.81)	0.799
Father of respondent	-	-	-	-

The respondents aged equal to or greater than 35 years were more likely (AOR 1.65, 95% CI; 1.06, 2.57) to have institutional delivery of their wives than those with the age equal to or less than 24 years. Men with graduate and above level of education were more likely (12.11, 95% CI; 7.64, 19.19) to have institutional delivery of their wives compared to uneducated or men with primary level of education. Men with higher household income (Q5) were more likely (2.74, 95% CI; 1.81, 4.15) to have institutional delivery of their wives than those of lower income (Q1). Men with knowledge about danger signs during pregnancy were more likely (11.06, 95% CI; 7.76, 15.76) to have institutional delivery of their wives than men with no knowledge. Interestingly, when the mother of respondent was decision maker for seeking maternal health at home were more likely (2.15, 95% CI; 1.02, 4.54) to have institutional delivery of their wives compared to decision made by their wives. The Men who had equal to or more than three children (0.27, 95% CI; 0.19, 0.38) and were non-indigenous ethnicities (0.70, 95% CI; 0.47, 1.04) were less likely to have institutional delivery of their wives than the men who hadequal to or less than two children and from indigenous ethnicities (Table 3).

**Table 3:** Paternal factors associated with institutional delivery of wife(N = 2178).

Characteristics	Crude odds ratio 95% CI	P-value	Adjusted odds ratio 95% CI	P-value
**Age**				
≤24 years	Reference		Reference	
25–34 years	0.94 (0.73, 1.20)	0.604	1.35 (0.92, 1.97)	0.124
≥35years	0.78 (0.60, 1.12)	0.067	1.65 (1.06, 2.57)	0.028
**Education**				
Illiterate & primary level	Reference		Reference	
Secondary to higher secondary level	11.5 (8.27, 16.00)	<0.001	5.93 (4.11, 8.55)	<0.001
Graduate & above level	92.0 (62.78, 134.83)	<0.001	12.11 (7.64, 19.19)	<0.001
**Income**				
Q1-poorest	Reference		Reference	
Q2	0.31 (0.24, 0.41)	<0.001	0.50 (0.34, 0.74)	0.001
Q3	1.02 (0.77, 1.34)	0.914	1.22 (0.80, 1.88)	0.354
Q4	1.14 (0.87, 1.50)	0.339	0.71 (0.47, 1.06)	0.092
Q5-richest	4.82 (3.61, 6.44)	<0.001	2.74 (1.81, 4.15)	<0.001
**Religion**				
Others (Buddhist, Christian, Muslim)	Reference		Reference	
Hindu	4.05 (2.97, 5.52)	<0.001	3.74 (2.22, 6.29)	<0.001
**Ethnicity**				
Indigenous	Reference		Reference	
Non-indigenous	2.10 (1.67, 2.65)	<0.001	0.70 (0.47, 1.04)	0.074
**Number of children**				
1–2 children	Reference		Reference	
≥ 3 children	0.19 (0.16, 0.24)	<0.001	0.27 (0.19, 0.38)	<0.001
**Knowledge of danger signs**				
No	Reference		Reference	
Yes	24.29 (17.99, 32.79)	<0.001	11.06 (7.76, 15.76)	<0.001
**Decision maker for MCH**				
Wife	Reference		Reference	
Husband	0.57 (0.37, 0.87)	0.010	1.87 (1.04,3.36)	0.036
Mother of respondent	0.37 (0.21, 0.65)	<0.001	2.15 (1.02,4.54)	0.045
Father of respondent	-	-	-	-

**Fig 1:**
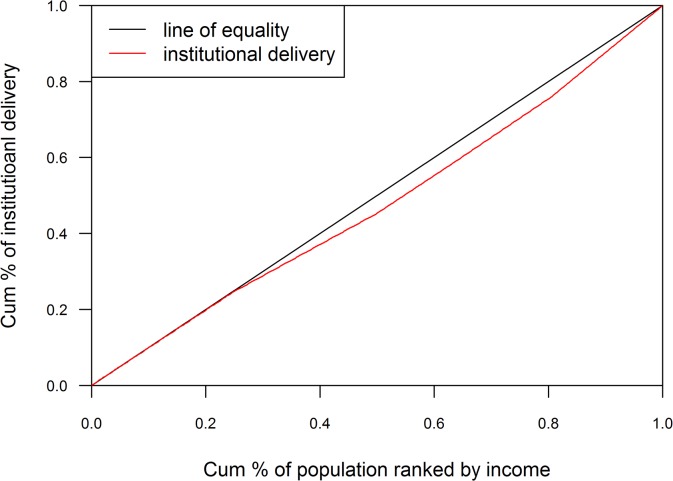
Place of delivery (Institutional).

**Fig 2:**
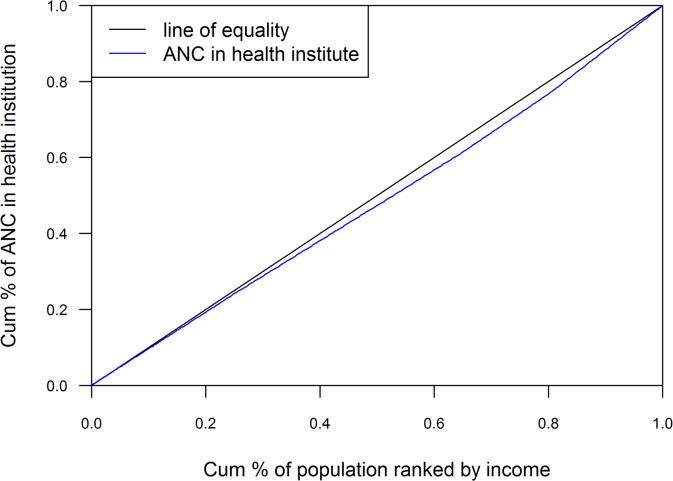
ANC (Institutional).

**Fig 3:**
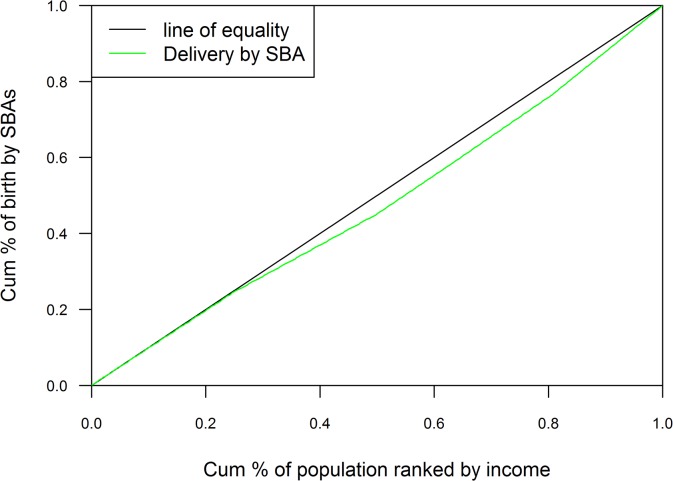
Births attend by SBAs.

The assessment of concentration curves for receiving institutional delivery, showed inequity in the use of service being disproportionately utilized more by rich people and less by poor people (Fig 1). A similar inspection was illustrated in Figs 2 and 3 with ANC and SBA respectively, showing greater use of services by rich respondents. Concentration index scenarios were depicted with respect to institutional delivery, ANC and SBA, which was 0.05, 0.04, and 0.05 respectively. The scenario of concentration index demonstrated great equity in service use.

### Predictive path model

**Fig 4:**
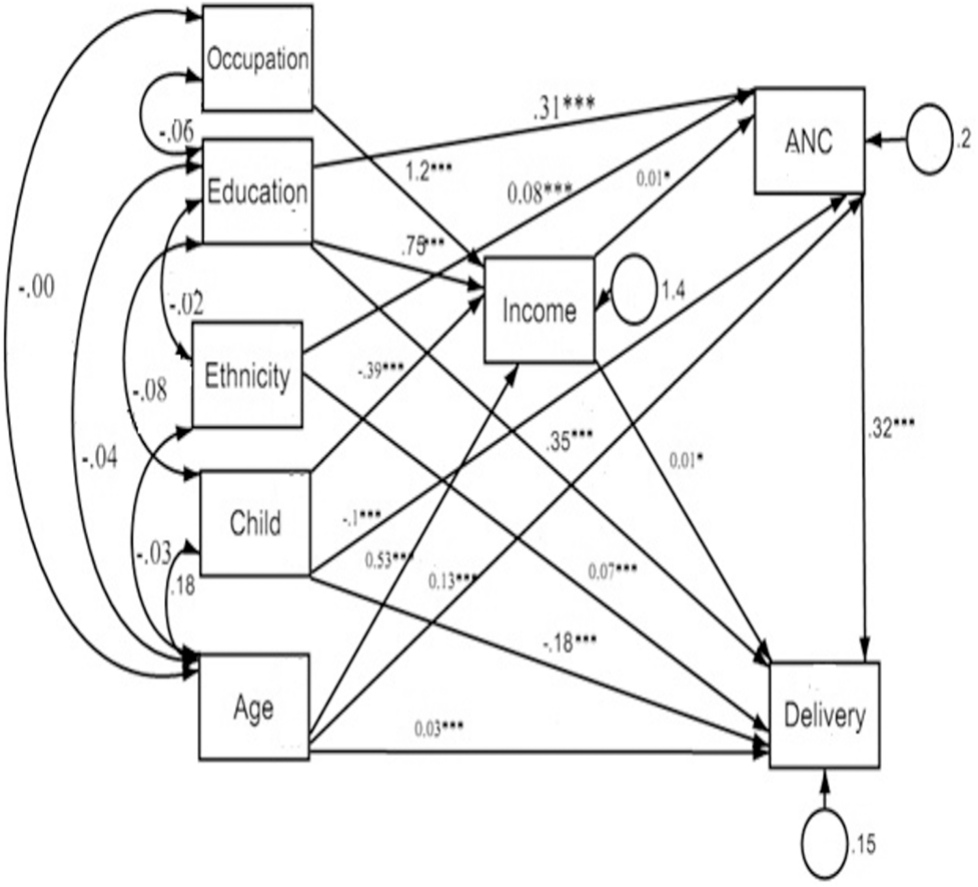
Significant regression paths among dependent and independent variables in the structural equation model (N = 2178). *p ≤.05, ***p ≤.001, Regression coefficients represented as one-way arrows.

Fig 4 shows the final structural equation model after finalization the model. Goodness-of-fit for the final path model was good: χ2 p-value was 0.72 for likelihood ratio, CFI = 1.00, TFI = 1.00 RMSEA = .000, CI = .00 to .03. The household income played an essential role in the association between ANC and institutional delivery. Income was statistically significant with ANC and institutional delivery. Similarly to their bivariate associations like education directly associated with more ANC visits and institutional delivery, and the number of children was associated with less ANC visits and institutional delivery. ANC was directly associated with the institutional delivery.

## Discussion

In this study, the associations between paternal factors and maternal health care services especially antenatal care and institutional delivery were examined. Any study conducted among male participants to get information on maternal and child health was not found. It was tried in this study to analyze the bi-directional causal effect among antenatal care and institutional delivery with the help of structural equation modeling. This paper tried to analyze inequities in the access to maternal health care services in Nepal based on a household income index. The results show that the economic variation can play an important role in the maternal health care service utilization. The findings reveal the inequality in the use of services which is distinct between the poor and the rich within the income quintiles.

The findings also show the association between respondent’s income quintiles and institutional delivery, and antenatal care. The finding on association of the utilization of maternal health care services with the crucial factor of richest income quintile is in similar agreement with the previous studies from developing and other countries [3,15–21,30–32]. This evidence suggests that income inequality is a crucial risk factor for the use of maternal health care services. Inequity gaps for maternal health care service utilization are extensively ongoing in developing countries including Nepal, which indicates that the services are not favorable for poor people.

Male are the key gate keeper in supporting women’s access to maternal health care services[27]. Education of husband might be an important factor in sustaining women’s access to use maternal health service utilization. The findings also reveal that the higher education is associated factors for maternal health service utilization. Previous finding also indicated that educated male had better knowledge about the benefit of service utilization, and it increases the awareness of service utilization[3,14,15,33]. Previous studies had indicated that education is an important associated factor with antenatal care and institutional delivery[3,32,34,35]. Our results suggested that the older aged male facilitated more to their wives to have access in maternal health services utilization. This might be due to increased awareness of maternal health with the increasing age of males. The results indicated that the higher the number of children, the lesser is the utilization is of institutional delivery and ANC. Traditional or conservative attitude and experience of multiple births might be the possible cause for lower utilization of institutional delivery. In addition, problem in transportation, lack of information and inadequate availability of health services might be the constraints for low utilization.

Previous studies had emphasized that the ethnicity was significantly associated with antenatal care and institutional delivery[3,19,20,32]. The findings of our study related to ethnicity and institutional delivery are contradictory with the previous finding. The finding from Nepal demographic health survey 2011 indicated that non-indigenous ethnic people had higher access in maternal health services[3]. Non-indigenous ethnic people are more traditional and conservative than indigenous in Nepal. Most of the women in Nepal regard their husbands as God and do not go against them. This traditional or socio-cultural practice might be the reason for less utilization of services among non-indigenous ethnic group. Our findings of this study have established the significant association between knowledge about danger signs during pregnancy and maternal health service utilization. Increased knowledge about danger signs during pregnancy could be a crucial factor when developing programs and policies. Finding of this study reveals that maternal health services are more utilized when a husband and the respondents’ mother is the decision maker for maternal health issues. The possible reason behind this might be the conservative traditional culture of society, and the lack of knowledge related to pregnancy, maternal and child health. Previous studies from Nepal, India and Bangladesh indicated that two fifth of male had active involvement on decision making for women’s health[3,36]. This could be similar in most of the developing countries where husband has all the decision making powers and there was bias[15,17].

Antenatal care helps to reduce risks associated with maternal deaths related to pregnancy complications [6]. Although, comparative contribution of ANC to maternal health is contradictory [6,7], a systematic review indicated an association between ANC and utilization of institutional delivery services [8]. Structural equation model of this study indicates that increasing ANC has positive significant effect on increasing utilization of institutional delivery. Motivational attitudes of health professionals and quality of service in ANC might be the encouragement for utilization of institutional delivery.

### Strengths and weaknesses of the study

In consideration, this study also had limitations: The information collected only from male subjects might not be complete and consistent information related to ANC and delivery. Information related to ANC and delivery was taken after the gap of those events could be bias. The detail information on household wealth was not collected. The information on respondent’s household income per month was used to develop a quintile index, failing to compute a wealth index of the population. Other contributing factors including education level of females, history of labour,media exposure, availability of health facilities, transportation facilities, and role of family member were not considered in this study which are crucial to develop policy and programs.

Strength of this study: The findings of this study are generalizable among both hospital based and community-based studies. Adequate sample size for this study also helped in making strong conclusions. The analysis for this study followed standard measures increasing the reliability of the outcomes.

## Conclusions

Paternal factors including age, household wealth, number of children, ethnicity, education, knowledge of danger sign during pregnancy and husband as decision maker for MCH are crucial factors associated with maternal health service utilization. Hence promoting male involvement in maternal health would yield better results in increasing the use of health services. Higher ANC coverage predicts higher utilization of the institutional delivery, therefore it is exceptionally significant to encourage the use of ANC. Maternal health services are concentrated among wealthier population and poor-rich inequities are necessary to be addressed through effective program and policy.
